# Prognostic value of pre-operative inflammatory response biomarkers in gastric cancer patients and the construction of a predictive model

**DOI:** 10.1186/s12967-015-0409-0

**Published:** 2015-02-18

**Authors:** Qiwen Deng, Bangshun He, Xian Liu, Jin Yue, Houqun Ying, Yuqin Pan, Huiling Sun, Jie Chen, Feng Wang, Tianyi Gao, Lei Zhang, Shukui Wang

**Affiliations:** Central Laboratory, Nanjing First Hospital, Nanjing Medical University, Nanjing, Jiangsu 210006 China; Department of Laboratory Medicine, Huai’an Second People’s Hospital, Huai’an, Jiangsu China; Medical college, Southeast University, Nanjing, Jiangsu China; Department of Life Sciences, Nanjing Normal University, Nanjing, Jiangsu China; Department of Laboratory Medicine, Nanjing First Hospital, Nanjing Medical University, Nanjing, Jiangsu China; Department of Orthopaedics, Lanxi People’s Hospital, Jinhua, Zhejiang China

**Keywords:** Inflammation, Gastric cancer, Prognosis, Survival, Nomogram

## Abstract

**Background:**

Inflammation plays an integral role in carcinogenesis and tumor progression. Inflammatory response biomarkers have shown to be promising prognostic factors for improving the predictive accuracy in various cancers. The aim of this study is to investigate the prognostic significance of pre-operative neutrophil to lymphocyte ratio (NLR), derived neutrophil to lymphocyte ratio (dNLR), platelet to lymphocyte ratio (PLR) and lymphocyte to monocyte ratio (LMR) in gastric cancer (GC).

**Methods:**

389 patients who had undergone gastrectomy were enrolled from 2007 to 2009 in this study. NLR, dNLR, PLR and LMR were calculated from peripheral blood cell count taken at pre-operation. Receiver operating curve (ROC) was used to determine the optimal cut-off levels for these biomarkers. A predictive model or nomogram was established to predict prognosis for cancer-specific survival (CSS) and disease-free survival (DFS), and the predictive accuracy of the nomogram was determined by concordance index (c-index).

**Results:**

The median follow-up period was 24 months ranging from 3 months to 60 months. The optimal cut-off levels were 2.36 for NLR, 1.85 for dNLR, 132 for PLR and 4.95 for LMR by ROC curves analysis. Elevated NLR, dNLR and PLR were significantly associated with worse overall survival (OS), CSS and DFS, however, elevated LMR showed an adverse effect on worse OS, CSS and DFS. Multivariate analysis revealed that elevated dNLR was an independent factor for worse OS, and NLR was superior to dNLR, PLR and LMR in terms of hazard ratio (HR = 1.53, 95% CI = 1.11-2.11, *P* = 0.010), which was shown to be independent prognostic indicators for both CSS and DFS. Moreover, the nomogram could more accurately predict CSS (c-index: 0.89) and DFS (c-index: 0.84) in surgical GC patients.

**Conclusions:**

Pre-operative NLR and dNLR may serve as potential prognostic biomarkers in patients with GC who underwent surgical resection. The proposed nomograms can be used for the prediction of CSS and DFS in patients with GC who have undergone gastrectomy.

## Introduction

Gastric cancer (GC) is one of the most common causes of cancer death worldwide [1,2], it is also the second most common cancer and the third leading cause of death among various types of cancer in China. Despite substantial progress achieved in diagnosis and treatment for GC in recent years, the overall 5-year survival rate remains unsatisfactory due to local relapses or metastasis after resection of primary GC. The survival rate will obviously be improved if the disease can be detected at an early tumor stage [3]. Because of the heterogeneity among the prognosis of GC, it is necessary to seek effective biomarkers for the early detection and prognosis prediction in patients with GC.

Recently, emerging evidence indicates that inflammation plays a critical role in the initiation and progression of numerous cancers, including GC [4,5]. About 15% of cancer-related deaths are closely associated with chronic inflammation or unresolved infection, and prolonged inflammation may lead to GC, hepatocellular carcinoma, and colorectal cancer [6]. Generally, systemic inflammatory response was the characteristic of changes in the relative levels of circulating white blood cells (WBC), largely resulting from the change of neutrophils and lymphocytes. Interestingly, cancer cells themselves can recruit and activate various leukocytes (especially neutrophils and monocytes) by T cells [7], specific chemokines [8,9] and prostaglandins [4]. The activation of platelet stimulated by proinflammatory cytokines participates in neutrophil recruitment and promotes inflammatory response [10]. The inflammatory response to tumor may contribute to tumor growth, progression and metastasis through several mechanisms, including the up-regulation of inflammatory mediators and cytokine, aberrant activation of immune regulatory cytokines, suppression of apoptosis, and DNA damage [6]. Those biomarkers in the peripheral blood that can exhibit the status of inflammation are considered as potential predictive markers for cancer prognosis.

In recent years, several indicators derived from the peripheral blood such as the neutrophil to lymphocyte ratio (NLR), derived neutrophil to lymphocyte ratio (dNLR), platelet to lymphocyte ratio (PLR) and lymphocyte to monocyte ratio (LMR) have been widely investigated as useful prognostic indicators in various kinds of cancers including GC, colorectal cancer, hepatocellular carcinoma, non-small cell lung cancer, breast cancer and pancreatic cancer [11-16]. However, only one or two inflammatory biomarkers have been evaluated for the prognosis of patients with GC according to previous reports [11,17-21]. Moreover, the optimal cut-off values of the biomarkers from these studies were still inconsistent.

Therefore, further study on the prognosis values of these biomarkers in patients with GC is necessary. This study aimed to evaluate the potential prognostic biomarkers for 389 patients with GC undergoing surgical resection, and was the first study attempting to establish a prognostic nomogram with improved predictive capacity for patients with GC based on these biomarkers and the clinicopathologic parameters.

## Patients and methods

### Patients

Medical records of all newly diagnosed GC patients between January 2007 and January 2009 in Nanjing First Hospital (Jiangsu, China) were collected and retrospectively analyzed in the present study. The diagnosis of GC was confirmed depended on histological evidence and classified based on the seventh edition of the TNM-UICC/AJCC classification [22]. The inclusion criteria were as follows: 1) All patients with GC underwent a total or subtotal gastrectomy with standard lymphadenectomy. 2) No prior pre-operation anticancer treatment, such as chemotherapy, or radiotherapy. 3) Post-operation estimated life expectancy ≥ 3 months. 4) No hematology disease, infection, hyperpyrexia and gastrobrosia. 5) Informed consents were obtained from eligible patients. At last, 389 patients were enrolled in the present study. This study was approved by Medical Ethics Committee of Nanjing First Hospital Affiliated to Nanjing Medical University.

### Blood sample analysis

All peripheral blood was collected in tubes containing ethylenediaminetetraacetic acid (EDTA) pre-operation. Blood cell counts were detected by Sysmex XT-1800i Automated Hematology System (Shanghai, China), including white blood cell, neutrophil, lymphocyte, platelet and monocyte counts. The concentrations of serum CEA and CA199 were measured using electrochemiluminescence by ELECSYS 2010 (Roche, Switzerland). Pre-operative neutrophil count to lymphocyte count (NLR), derived neutrophil to lymphocyte ratio (dNLR), platelet to lymphocyte ratio (PLR) and lymphocyte to monocyte ratio (LMR) were calculated from peripheral blood cell count.

### Patient follow-up

After operation, each patient was followed up regularly until January 2014 or until death (every 3 months for the first 2 years and then every 6 months up to 5th year). Physical examination, endoscopy, laboratory tests and imaging were conducted at every visit. The follow-up periods varied from 3 months to 60 months, with a median of 24 months. Overall survival (OS) was calculated from surgery to death. For drop-out patients, the date of the last follow-up was applied. Disease-free survival (DFS) was calculated from surgery to disease relapse or until the date of last follow-up. Cancer specific survival (CSS) was calculated from surgery to cancer-related death.

### Statistical analysis

The optimal cut-off levels of NLR, dNLR, PLR, and LMR were determined by receiver operating curve (ROC) analysis [23,24]. The end-point based on CSS, OS or DFS was also applied to achieve the optimal cut-off points. Our results showed that the optimal cut-off levels were similar in these biomarkers (Table 1). Considering some previous studies employed CSS as the end-point to calculate the optimal cut-off point [24-26]. The optimal cut-off levels were calculated by the end-point based on CSS in the present study. Chi-square test or Fisher’s exact test was used to compare categorical variables, and Mann–Whitney *U* or Kruskal-Wallis test was applied to compare continuous variables between groups. Continuous variables were expressed as mean ± SD. Survival rates were calculated by Kaplan-Meier survival analysis and the significance was evaluated by Log-rank test. The predictors for on OS, DFS and CSS determined by univariate analysis were evaluated by multivariate analysis using Cox’s proportional hazards model. Nomograms for DFS and CSS were established by R 3.0.3 software (Institute for Statistics and Mathematics, Vienna, Austria), and the predictive accuracy was evaluated by Harrell’s concordance index (c-index). All statistical analyses were conducted using IBM SPSS 20.0 software (IBM, USA). *P* values less than 0.05 were considered statistically significant.

**Table 1:** **The optimal cut-off point based on CSS, OS and DFS as end-point**

**Variables**	**CSS**		**OS**		**DFS**	
	**AUC**	**Cut-off point**	**AUC**	**Cut-off point**	**AUC**	**Cut-off point**
NLR	0.703	2.36	0.692	2.46	0.680	2.46
dNLR	0.683	1.85	0.676	1.85	0.665	1.81
PLR	0.585	132	0.579	132	0.588	123
LMR	0.695	4.95	0.674	4.92	0.660	5.12

NLR, neutrophil count to lymphocyte count; dNLR, neutrophil count to (white cell count – neutrophil count); PLR, platelet count to lymphocyte count; CSS, cancer-specific survival; OS, overall survival; DFS, disease-free survival; AUC, area under curve.

## Results

### Clinicopathologic characteristics of patients

389 GC patients with 282 (72.5%) male and 107 (27.5%) female patients aged from 29 to 92 (the median age was 65 years). 15 patients had a family history of GC. The median follow-up period was 24 months. According to histological type, the number of papillary, tubular, poorly differentiated, mucinous and signetring cell carcinoma were 19 (4.9%), 81 (20.8%), 203 (52.2%), 57 (14.7%) and 29 (7.4%), respectively. Based on the seventh edition of the TNM-UICC/AJCC classification, the number of stage I-II and III-IV were 159 (40.9%) and 230 (59.1%), respectively. The number of patients with tumor grade G1-G2 and G3-G4 was 214 (55.0%) and 175 (45.0%), respectively. During the follow-up period, 302 (77.6%) patients were detected as local recurrence or distant metastasis. Among them, 270 (69.4%) patients were dead with 235 (60.2%) patients died from cancer-related disease. The median of DFS and OS was 18 months and 24 months, respectively. In addition, the median values of serum CEA, CA199, NLR, dNLR, PLR and LMR were 3.16 (0.2-500) ng/ml, 12.21 (0.01-1000) U/ml, 3.19 (0.56-74.49), 2.27 (0.37-45.64), 145 (5.37-3111) and 3.97 (0.52-71.67), respectively.

### The optimal thresholds for NLR, dNLR, PLR and LMR

**Figure 1:**
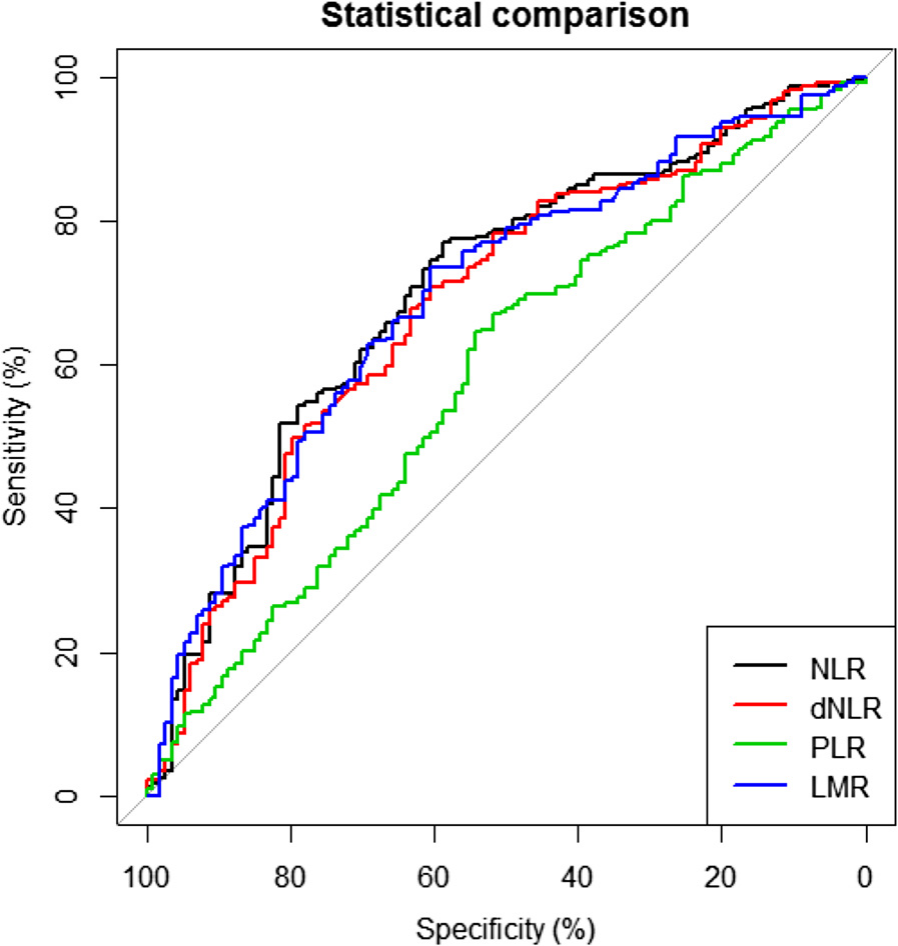
**Optimal cut-off levels for NLR, dNLR, PLR and LMR were applied with ROC curves for cancer-specific survival.**

The ROC curves, using CSS as the end-point for NLR, dNLR, PLR and LMR, were depicted in Figure 1. The areas under curve (AUC) for NLR, dNLR, PLR and LMR were 0.703 (*P* = 0.000), 0.683 (*P* = 0.000), 0.585(*P* = 0.010) and 0.695 (*P* = 0.000), respectively. The optimal cut-off levels based on CSS were determined to be 2.36 for NLR, 1.85 for dNLR, 132 for PLR and 4.95 for LMR by ROC curves analysis. Patients were subsequently divided into two groups according to the optimal cut-off levels, with the high group ≥ the optimal cut-off levels and the low group that < the optimal cut-off levels.

### The correlation of NLR, dNLR, PLR and LMR with the clinicopathologic parameters

**Figure 2:**
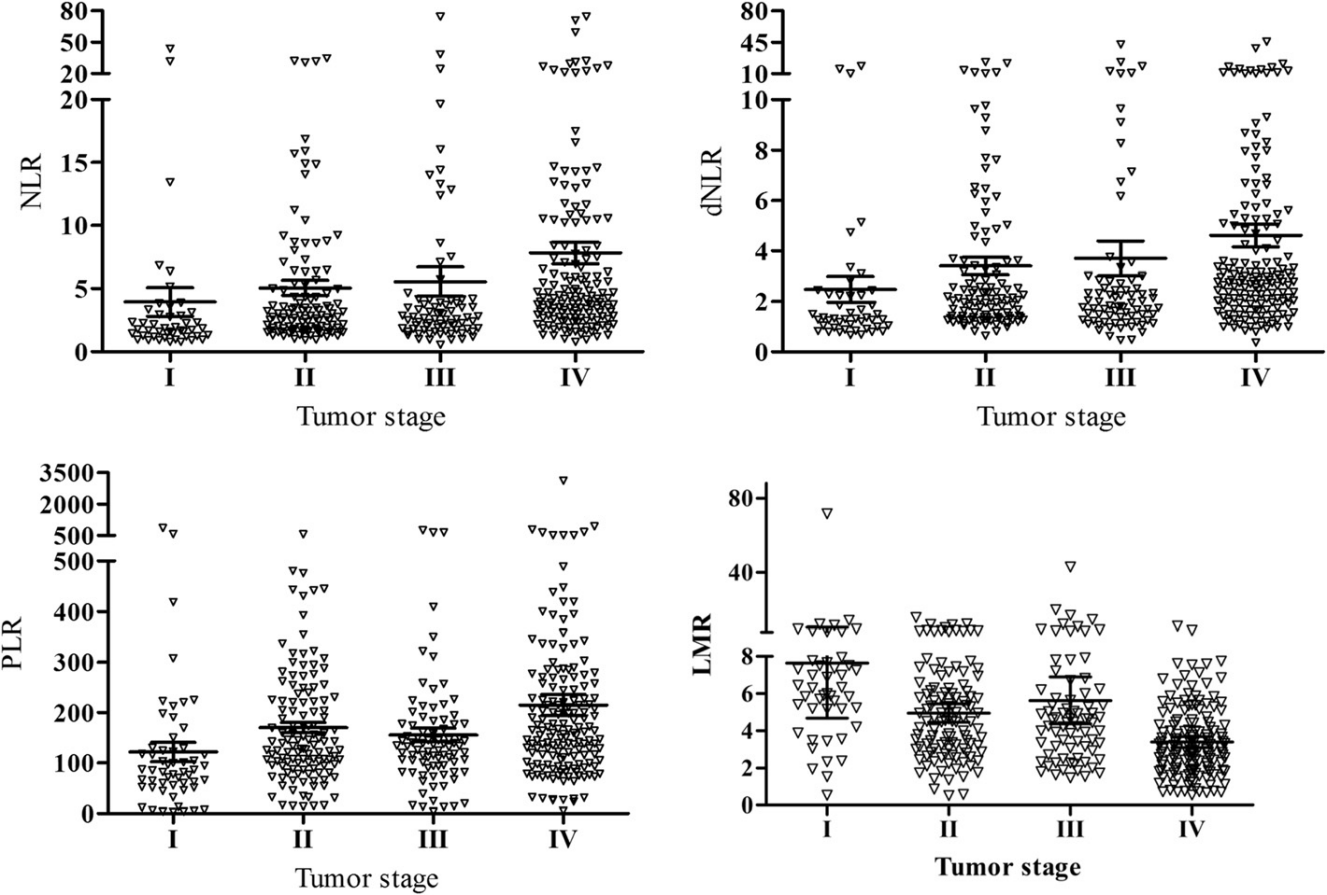
**The relationship between NLR, dNLR, PLR, LMR and tumor stage in patients with gastric cancer.**

To study the correlation of NLR, dNLR, PLR and LMR with clinicopathologic parameters, comparison between the high and low groups for NLR, dNLR, PLR and LMR was carried out (Table 2; Figure 2). Our results revealed that NLR, dNLR, PLR and LMR were significantly correlated with tumor stage, depth of invasion, lymph node, distant metastasis, CEA and CA199 (*P*_all_ < 0.05), separately. Moreover, NLR, dNLR and LMR were closely associated with the age of patients, and PLR had significant correlation with the sex of patients and histological types. Those patients with tumor stage III-IV, depth of invasion T3-T4, lymph node N1-N3 and distant metastasis M1 had a higher NLR, dNLR and PLR than those with tumor stage I-II, depth of invasion T1-T2, lymph node N0 and distant metastasis M0 (Table 3). However, no significant association was observed between tumor grade and the inflammatory biomarkers (NLR, dNLR, PLR and LMR). Intriguingly, GC patients had a lower LMR in tumor stage (III-IV), depth of invasion (T3-T4), lymph node (N1-N3) and distant metastasis (M1) (*P*_all_ < 0.05, Table 3).

**Table 2:** **Baseline patient characteristics according to total patients, NLR, dNLR, PLR and LMR level**

**Characteristics**		**Total patients (n = 389)**	**Patients grouped by NLR level (n = 389)**		***P*** *	**Patients grouped by dNLR level (n = 389)**		***P*** *	**Patients grouped by PLR level (n = 389)**		***P*** *	**Patients grouped by LMR level (n = 389)**		***P*** *
			**NLR < 2.36 (n = 132)**	**NLR ≥ 2.36 (n = 257)**		**dNLR < 1.85 (n = 151)**	**dNLR ≥ 1.85 (n = 238)**		**PLR < 132 (n = 165)**	**PLR ≥ 132 (n = 224)**		**LMR < 4.95 (n = 241)**	**LMR ≥ 4.95 (n = 148)**	
Sex	Female	107 (27.5%)	37 (28.0%)	70 (27.2%)	0.868	41 (27.2%)	66 (27.7%)	0.901	54 (32.7%)	53 (23.7%)	**0.048**	59 (24.5%)	48 (32.4%)	0.088
	Male	282 (72.5%)	95 (72.0%)	187 (72.8%)		110 (72.8%)	172 (72.3%)		111 (67.3%)	171 (76.3%)		182 (75.5%)	100 (67.6%)	
Age (years)	<65	185 (47.6%)	74 (56.1%)	111 (43.2%)	**0.016**	83 (55.0%)	102 (42.9%)	**0.020**	87 (52.7%)	98 (43.8%)	0.080	98 (40.7%)	87 (58.8%)	**0.001**
	≥65	204 (52.4%)	58 (43.9%)	146 (56.8%)		68 (45.0%)	136 (57.1%)		78 (47.3%)	126 (56.2%)		143 (59.3%)	61 (41.2%)	
Alcohol	Yes	73 (18.8%)	31 (23.5%)	42 (16.3%)	0.088	32 (21.2%)	41 (17.2%)	0.329	36 (21.8%)	37 (16.5%)	0.186	44 (18.3%)	29 (19.6%)	0.743
	No	316 (81.2%)	101 (76.5%)	215 (83.7%)		119 (78.8%)	197 (82.8%)		129 (78.2%)	187 (83.5%)		197 (81.7%)	119 (80.4%)	
Tobacco	Yes	72 (18.5%)	29 (22.0%)	43 (16.7%)	0.208	30 (19.9%)	42 (17.6%)	0.583	37 (22.4%)	35 (15.6%)	0.088	46 (19.1%)	26 (17.6%)	0.708
	No	317 (81.5%)	103 (78.0%)	214 (83.3%)		121 (80.1%)	196 (82.4%)		128 (77.6%)	189 (84.4%)		195 (80.9%)	122 (82.4%)	
Family history	Yes	15 (3.9%)	4 (3.0%)	11 (4.3%)	0.544	5 (3.3%)	10 (4.2%)	0.657	9 (5.5%)	6 (2.7%)	0.160	6 (2.5%)	9 (6.1%)	0.100
	No	374 (96.1%)	128 (97.0%)	246 (95.7%)		146 (96.7%)	228 (95.8%)		156 (94.5%)	218 (97.3%)		235 (97.5%)	139 (93.9%)	
Histological type	papillary	19 (4.9%)	8 (6.1%)	11 (4.3%)	0.543	10 (6.7%)	9 (3.8%)	0.424	15 (9.1%)	4 (1.8%)	**0.009**	9 (3.7%)	10 (6.8%)	0.260
	tubular	81 (20.8%)	26 (19.7%)	55 (21.4%)		32 (21.1%)	49 (20.6%)		30 (18.2%)	51 (22.8%)		45 (18.7%)	36 (24.3%)	
poorly differentiated		203 (52.2%)	66 (50.0%)	137 (53.3%)		74 (49.0%)	129 (54.2%)		79 (47.9%)	124 (55.4%)		135 (56.0%)	68 (45.9%)	
	mucinous	57 (14.7%)	24 (18.1%)	33 (12.8%)		26 (17.2%)	31 (13.0%)		28 (16.9%)	29 (12.9%)		33 (13.7%)	24 (16.2%)	
signetring cell		29 (7.4%)	8 (6.1%)	21 (8.2%)		9 (6.0%)	20 (8.4%)		13 (7.9%)	16 (7.1%)		18 (7.5%)	11 (7.4%)	
Tumor grade	G1-G2	214 (55.0%)	68 (51.5%)	146 (56.8%)	0.320	78 (51.7%)	136 (58.0%)	0.289	85 (51.5%)	129 (57.6%)	0.234	133 (55.2%)	81 (54.7%)	0.930
	G3-G4	175 (45.0%)	64 (48.5%)	111 (43.2%)		73 (48.3%)	102 (42.0%)		80 (48.5%)	95 (42.4%)		108 (44.8%)	67 (45.3%)	
Tumor stage	I-II	159 (40.9%)	95 (72.0%)	64 (24.9%)	**0.000**	78 (51.7%)	81 (34.0%)	**0.001**	83 (50.3%)	76 (33.9%)	**0.001**	72 (29.9%)	87 (58.8%)	**0.000**
	III-IV	230 (59.1%)	37 (28.0%)	193 (75.1%)		73 (48.3%)	157 (66.0%)		82 (49.7%)	148 (66.1%)		169 (70.1%)	61 (41.2%)	
Depth of invasion	T1-T2	66 (17.0%)	38 (28.8%)	28 (10.9%)	**0.000**	37 (24.5%)	29 (12.2%)	**0.002**	44 (26.7%)	22 (9.8%)	**0.000**	22 (9.1%)	44 (29.7%)	**0.000**
	T3-T4	323 (83%)	94 (71.2%)	229 (89.1%)		114 (75.5%)	209 (87.8%)		121 (73.3%)	202 (90.2%)		219 (90.9%)	104 (70.3%)	
Lymph node	N0	72 (18.5%)	41 (31.1%)	31 (12.1%)	**0.000**	42 (27.8%)	30 (12.6%)	**0.000**	43 (26.1%)	29 (12.9%)	**0.000**	27 (11.2%)	45 (30.4%)	0.000
	N1-N3	317 (81.5%)	91 (68.9%)	226 (87.9%)		109 (72.2%)	208 (87.4%)		122 (73.9%)	195 (87.1%)		214 (88.8%)	103 (69.6%)	
Distant metastasis	M0	235 (60.4%)	106 (80.3%)	129 (50.2%)	**0.000**	116 (76.8%)	119 (50.0%)	**0.000**	116 (70.3%)	119 (53.1%)	**0.001**	119 (49.4%)	116 (78.4%)	**0.000**
	M1	154 (39.6%)	26 (19.7%)	128 (49.8%)		35 (23.2%)	119 (50.0%)		49 (29.7%)	105 (46.9%)		122 (50.6%)	32 (21.6%)	
CEA (ng/ml)	≤5	243 (62.5%)	92 (69.7%)	151 (58.8%)	**0.034**	107 (70.9%)	136 (57.1%)	**0.006**	113 (68.5%)	130 (58.0%)	**0.035**	134 (55.6%)	109 (73.6%)	**0.000**
	>5	146 (37.5%)	40 (30.3%)	106 (41.2%)		44 (29.1%)	102 (42.9%)		52 (31.5%)	94 (42.0%)		107 (44.4%)	39 (26.4%)	
CA199 (U/ml)	≤37	278 (71.5%)	109 (82.6%)	169 (65.8%)	**0.001**	123 (81.5%)	155 (65.1%)	**0.001**	130 (78.8%)	148 (66.1%)	**0.006**	160 (66.4%)	118 (79.7%)	**0.005**
	>37	111 (28.5%)	23 (17.4%)	88 (34.2%)		28 (18.5%)	83 (34.9%)		35 (21.2%)	76 (33.9%)		81 (33.6%)	30 (20.3%)	

NLR, neutrophil count to lymphocyte count; dNLR, neutrophil count to (white cell count – neutrophil count); PLR, platelet count to lymphocyte count; LMR, lymphocyte to monocyte ratio.

*Difference between groups was tested by Chi-square test.

^a^Grade 1 and 2 stand for high or middle differentiated tumor, grade 3 and 4 stand for poorly differentiated tumor.

^b^Tumor stage according to seventh edition of American Joint of Committee on Cancer.

**Table 3:** **The association between patients’ pathological data and inflammatory biomarkers**

**Variables**	**Tumor grade** ^**a**^		***P*** *	**Tumor stage** ^**b**^		***P*** *	**Depth of invasion**		***P*** *	**Lymph node**		***P*** *	**Distant metastasis**		***P*** *
	**G1-G2**	**G3-G4**		**I-II**	**III-IV**		**T1-T2**	**T3-T4**		**N0**	**N1-N3**		**M0**	**M1**	
NLR	6.51 ± 10.79	5.64 ± 6.81	0.512	4.73 ± 6.73	7.07 ± 10.50	**0.000**	4.02 ± 6.67	6.54 ± 9.60	**0.000**	4.81 ± 8.03	6.41 ± 9.45	**0.000**	5.00 ± 7.92	7.82 ± 10.70	**0.000**
dNLR	4.08 ± 5.94	3.51 ± 3.61	0.516	3.13 ± 3.63	4.31 ± 5.76	**0.000**	2.67 ± 3.24	4.06 ± 5.29	**0.000**	3.01 ± 3.92	4.01 ± 5.23	**0.000**	3.32 ± 4.51	4.61 ± 5.65	**0.013**
PLR	210 ± 248	169 ± 103	0.185	170 ± 121	207 ± 235	**0.010**	146 ± 135	201 ± 206	**0.039**	160 ± 137	199 ± 208	**0.001**	170 ± 122	225 ± 272	**0.001**
LMR	4.65 ± 3.73	4.95 ± 5.83	0.808	5.74 ± 5.93	4.13 ± 3.66	**0.001**	6.90 ± 8.55	4.36 ± 3.42	**0.000**	6.71 ± 8.31	4.35 ± 3.40	**0.000**	5.70 ± 5.79	3.39 ± 1.88	**0.000**

NLR, neutrophil count to lymphocyte count; dNLR, neutrophil count to (white cell count – neutrophil count); PLR, platelet count to lymphocyte count; LMR, lymphocyte to monocyte ratio

*Difference between groups was tested by Mann–Whitney U or Kruskal-Wallis test.

^a^Grade 1 and 2 stand for high or middle differentiated tumor, grade 3 and 4 stand for poorly differentiated tumor.

^b^Tumor stage according to seventh edition of American Joint of Committee on Cancer.

### The association between baseline characteristics and clinical prognosis

**Figure 3:**
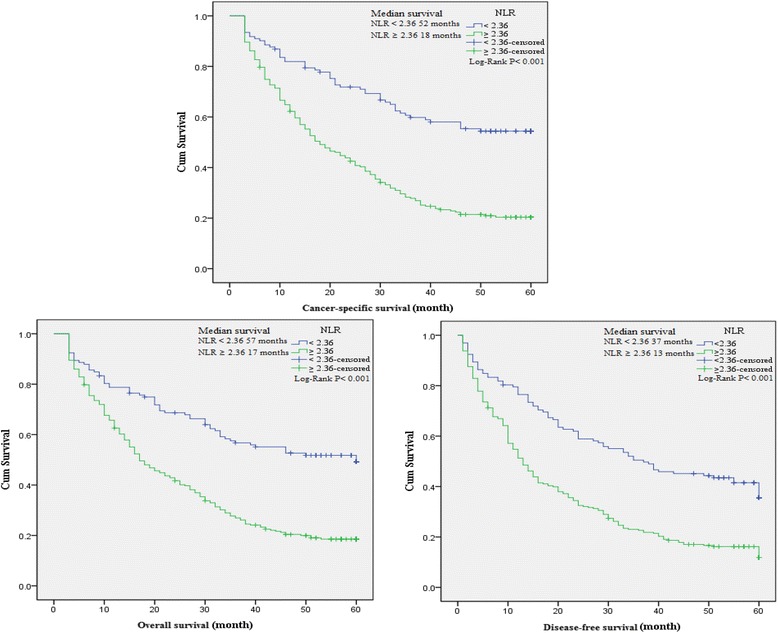
**Kaplan-Meier curves for overall, cancer-specific and disease-free survival probability according to NLR, dNLR, PLR and LMR level.** Patients with NLR ≥ 2.36 had a significantly associated with worse overall, cancer-specific and disease-free survival than those with NLR < 2.36.

**Figure 4:**
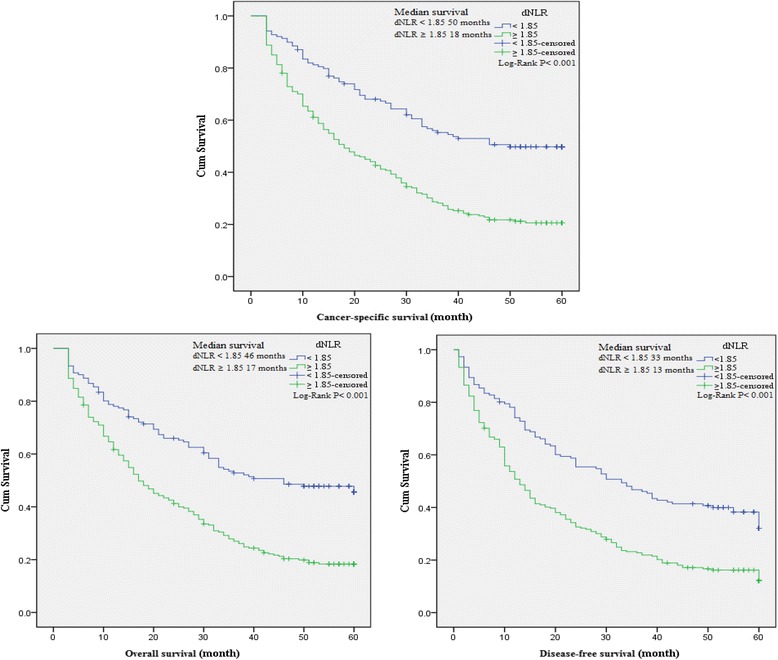
**Kaplan-Meier curves for overall, cancer-specific and disease-free survival probability according to NLR, dNLR, PLR and LMR level.** Overall, cancer-specific and disease-free survival with dNLR ≥ 1.85 was shorter than those with dNLR < 1.85.

**Figure 5:**
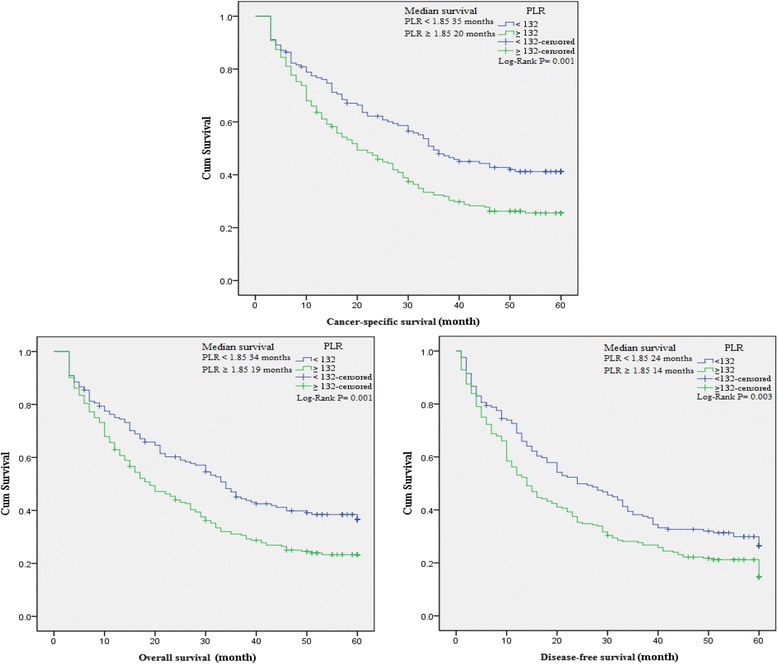
**Kaplan-Meier curves for overall, cancer-specific and disease-free survival probability according to NLR, dNLR, PLR and LMR level.** There was a significantly shorter overall, cancer-specific and disease-free survival in the group with PLR ≥ 132 than the group with PLR < 132.

**Figure 6:**
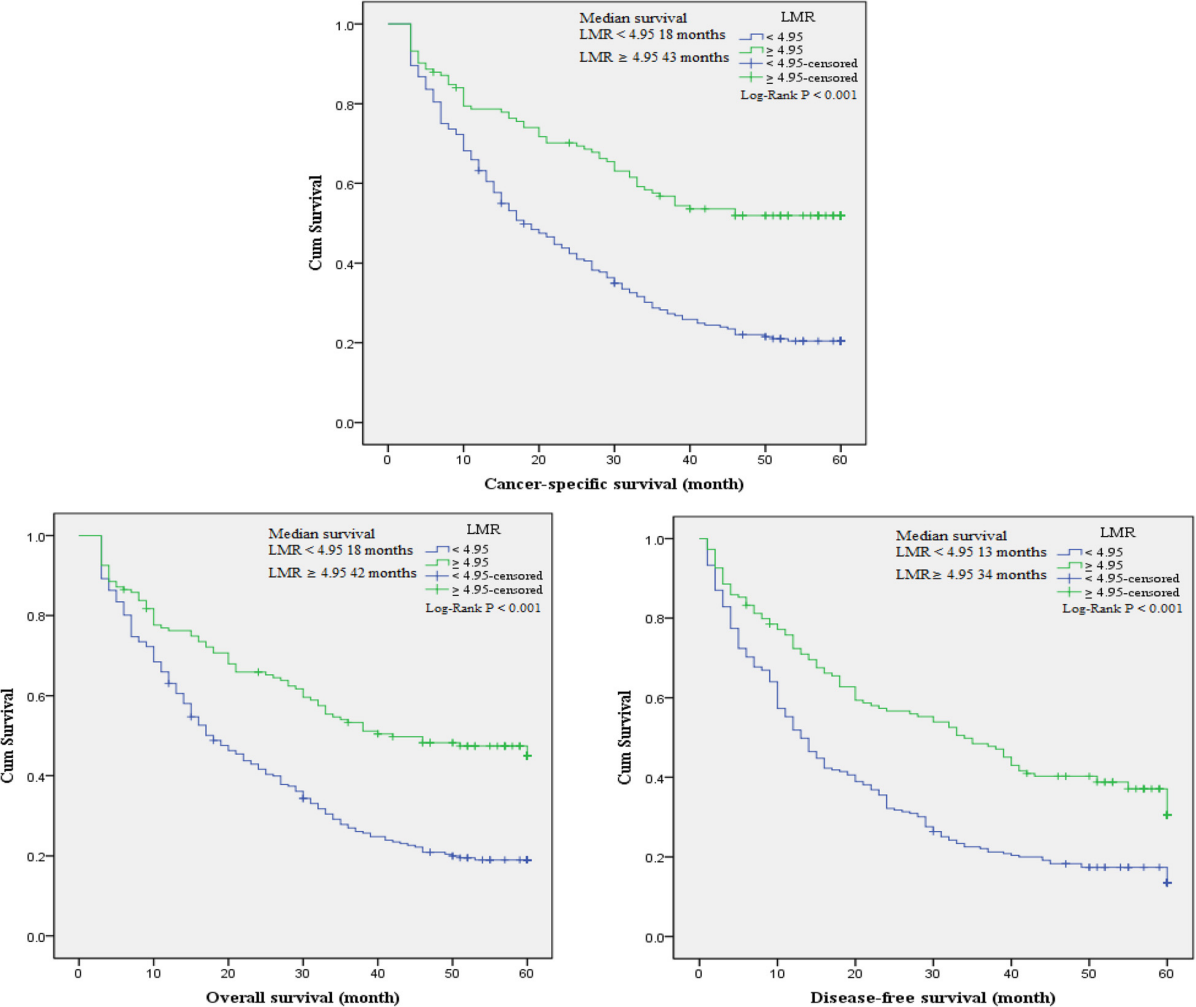
**Kaplan-Meier curves for overall, cancer-specific and disease-free survival probability according to NLR, dNLR, PLR and LMR level.** Patients with LMR ≥ 4.95 showed a longer overall, cancer-specific and disease-free survival than those with LMR < 4.95.

To evaluate the association of baseline characteristics with clinical prognosis, Kaplan-Meier survival analysis and log-rank tests were performed based on the postoperative survival time and the pathological data of patients. Our results indicated that NLR (≥2.36), dNLR (≥1.85), PLR (≥132), LMR (<4.95), age of patients (≥65 years), tumor stage III-IV, depth of invasion T3-T4, lymph node N1-N3, distant metastasis M1, CEA (>5 ng/ml) and CA199 (>37 U/ml) were significantly associated with decreased OS, CSS and DFS, and GC family history of patients was also associated with decreased DFS as well as histological types (Table 4). In addition, a significant relationship between the inflammatory biomarkers (NLR, dNLR, PLR and LMR) and clinical prognosis (OS, CSS and DFS) was observed (Figures 3, 4, 5, and 6).

**Table 4:** **The association of clinicopathological parameters with overall, cancer-specific and disease-free survival in patients**

**Characteristics**		**Total**	**Overall survival**	P*	**Cancer-specific survival**	P*	**Disease-free survival**	P*
		**n(%) 389**	**n(%) of patients with death (n = 270)**		**n(%) of patients with death (n = 235)**		**n(%) of patients with recurrence (n = 302)**	
Sex	Female	107 (27.5%)	72 (26.7%)	0.518	66 (28.1%)	0.629	80 (26.5%)	0.279
	Male	282 (72.5%)	198 (73.3%)		169 (71.9%)		222 (73.5%)	
Age (years)	<65	185 (47.6%)	111 (41.1%)	0.000	98 (41.7%)	0.000	132 (43.7%)	0.001
	≥65	204 (52.4%)	159 (58.9%)		137 (58.3%)		170 (56.3%)	
Alcohol	Yes	73 (18.8%)	48 (17.8%)	0.437	43 (18.3%)	0.439	55 (18.2%)	0.601
	No	316 (81.2%)	222 (82.2%)		192 (81.7%)		247 (81.8%)	
Tobacco	Yes	72 (18.5%)	56 (20.7%)	0.152	49 (20.9%)	0.125	61 (20.2%)	0.326
	No	317 (81.5%)	214 (79.3%)		186 (79.1%)		241 (79.8%)	
Family history	Yes	15 (3.9%)	9 (3.3%)	0.557	8 (3.4%)	0.554	14 (4.6%)	0.005
	No	374 (96.1%)	261 (96.7%)		227 (96.6%)		288 (95.4%)	
Histological type	papillary	19 (4.9%)	10 (3.7%)	0.163	9 (3.8%)	0.249	11 (3.6%)	0.041
	tubular	81 (20.8%)	53 (19.6%)		45 (19.1%)		59 (19.5%)	
poorly differentiated		203 (52.2%)	148 (54.8%)		126 (53.6%)		165 (54.6%)	
	mucinous	57 (14.7%)	37 (13.7%)		35 (14.9%)		43 (14.2%)	
signetring cell		29 (7.4%)	22 (8.1%)		20 (8.5%)		24 (7.9%)	
Tumor grade	G1-G2	214 (55.0%)	139 (51.5%)	0.061	127 (54.0%)	0.168	159 (52.6%)	0.044
	G3-G4	175 (45.0%)	131 (48.5%)		108 (46.0%)		143 (47.4%)	
Tumor stage	I-II	159 (40.9%)	60 (22.2%)	0.000	52 (22.1%)	0.000	85 (28.1%)	0.000
	III-IV	230 (59.1%)	210 (77.8%)		183 (77.9%)		217 (71.9%)	
Depth of invasion	T1-T2	66 (17.0%)	18 (6.7%)	0.000	16 (6.8%)	0.000	35 (11.6%)	0.000
	T3-T4	323 (83%)	252 (93.3%)		219 (93.2%)		267 (88.4%)	
Lymph node	N0	72 (18.5%)	17 (6.3%)	0.000	15 (6.4%)	0.000	34 (11.3%)	0.000
	N1-N3	317 (81.5%)	253 (93.7%)		220 (93.6%)		268 (88.7%)	
Distance metastasis	M0	235 (60.4%)	121 (44.8%)	0.000	106 (45.1%)	0.000	150 (49.7%)	0.000
	M1	154 (39.6%)	149 (55.2%)		129 (54.9%)		152 (50.3%)	
CEA (ng/ml)	≤5	243 (62.5%)	149 (55.2%)	0.000	131 (55.7%)	0.000	176 (58.3%)	0.000
	>5	146 (37.5%)	121 (44.8%)		104 (44.3%)		126 (41.7%)	
CA199 (U/ml)	≤37	278 (71.5%)	175 (64.8%)	0.000	151 (64.3%)	0.000	203 (67.2%)	0.000
	>37	111 (28.5%)	95 (35.2%)		84 (35.7%)		99 (32.8%)	
NLR	<2.36	132 (33.9%)	64 (23.7%)	0.000	54 (23.0%)	0.000	81 (26.8%)	0.000
	≥2.36	257 (66.1%)	206 (76.3%)		181 (77.0%)		221 (73.2%)	
dNLR	<1.85	151 (38.8%)	79 (29.3%)	0.000	68 (28.9%)	0.000	98 (32.5%)	0.000
	≥1.85	238 (61.2%)	191 (70.7%)		167 (71.1%)		204 (67.5%)	
PLR	<132	165 (42.4%)	100 (37.0%)	0.001	83 (35.3%)	0.001	117 (38.7%)	0.002
	≥132	224 (57.6%)	170 (63.0%)		152 (64.7%)		185 (61.3%)	
LMR	<4.95	241 (71.0%)	192 (71.1%)	0.000	173 (73.6%)	0.000	205 (67.9%)	0.000
	≥4.95	148 (29.0%)	78 (29.9%)		62 (26.4%)		97 (32.1%)	

NLR, neutrophil count to lymphocyte count; dNLR, neutrophil count to (white cell count – neutrophil count); PLR, platelet count to lymphocyte count; LMR, lymphocyte to monocyte ratio.

*Difference between two groups was tested by Kaplan-Meier survival analysis and Log-rank test.

^a^Grade 1 and 2 stand for high or middle differentiated tumor, grade 3 and 4 stand for poorly differentiated tumor.

^b^Tumor stage according to seventh edition of American Joint of Committee on Cancer.

Clinicopathological parameters for the prediction of clinical prognosis were further investigated by univariate analysis with Cox regression model. The significant parameters in univariate analysis combined with sex and age were then used to determine the influence on OS, CSS and DFS by multivariate analysis. Our results revealed that age, tumor stage, lymph node and distant metastasis were significantly associated with reduced OS and CSS (*P*_all_ < 0.01; Table 5). More importantly, elevated NLR (HR = 1.53, *P* = 0.010) and dNLR (HR = 1.42, *P* = 0.012) were also associated with reduced CSS and OS, respectively. Meanwhile, NLR (HR = 1.38, *P* = 0.025), GC family history (HR = 2.13, *P* = 0.007), tumor stage (HR = 2.04, *P* = 0.000), distant metastasis (HR = 2.06, *P* = 0.000) and CA199 (HR = 1.33, *P* = 0.028) were considered to be independent indicators for DFS of GC patients.

**Table 5:** **Univariate and multivariate analysis of prognostic factors of overall, cancer-specific and disease-free survival by Cox regression model**

**Variables**	**Overall survival**				**Cancer-specific survival**					**Disease-free survival**		
	**Univariate analysis**		**Multivariate analysis**		**Univariate analysis**		**Multivariate analysis**		**Univariate analysis**		**Multivariate analysis**	
	**HR**	***p***	**HR (95% CI)**	***p***	**HR**	***p***	**HR (95% CI)**	***p***	**HR**	***p***	**HR (95% CI)**	***p***
Sex (male)	0.92	0.525			0.93	0.634			0.87	0.290		
Age (≥65 years)	1.7	**0.000**	1.41 (1.10-1.80)	**0.007**	1.73	**0.000**	1.51 (1.16-1.96)	**0.002**	1.48	**0.001**	1.19 (0.94-1.50)	0.146
Alcohol (yes)	0.89	0.445			0.88	0.446			0.93	0.609		
Tobacco (yes)	1.24	0.160			1.27	0.133			1.15	0.336		
Family history (yes)	1.22	0.564		0.81	0.561	0.561			2.09	0.007	2.13 (1.23-3.69)	**0.007**
Histological type												
Papillary	1		1		1				1			
Non-papillary	1.83	0.061			1.77	0.093			1.89	**0.038**	1.42 (0.77-2.62)	0.260
Tubular	1.54	0.213			1.51	0.263			1.58	0.166		
Poorly differentiated	1.93	**0.045**	1.66 (0.86-3.19)	0.129	1.88	0.069			2.06	**0.020**	1.62 (0.88-3.01)	0.124
Mucinous	1.72	0.127			1.68	0.166			1.66	0.135		
Signetring cell	2.39	**0.022**	1.78 (0.83-3.79)	0.137	2.09	0.066			2.33	**0.021**	1.91 (0.93-3.91)	0.078
Tumor grade (G3-G4)	1.25	0.066			1.19	0.175			1.26	**0.049**	1.19 (0.93-1.50)	0.162
Tumor stage (III-IV)	4.87	**0.000**	2.18 (1.48-3.22)	**0.000**	5.45	**0.000**	2.46 (1.62-3.72)	**0.000**	3.58	**0.000**	2.04 (1.46-2.84)	**0.000**
Depth of invasion (T3-T4)	4.58	**0.000**	1.22 (0.67-2.22)	0.511	4.78	**0.000**	1.18 (0.63-2.21)	0.597	2.48	**0.000**	0.81 (0.50-1.32)	0.405
Lymph node (N1-N3)	5.6	**0.000**	2.34 (1.35-4.05)	**0.003**	5.36	**0.000**	2.12 (1.21-3.74)	**0.009**	3.05	**0.000**	1.39 (0.91-2.14)	0.132
Distant metastasis (M1)	4.61	**0.000**	2.14 (1.56-2.93)	**0.000**	5.07	**0.000**	2.09 (1.50-2.92)	**0.000**	3.73	**0.000**	2.06 (1.52-2.81)	0.000
CEA (>5 ng/ml)	1.66	**0.000**	0.87 (0.67-1.14)	0.323	1.74	**0.000**	0.93 (0.70-1.24)	0.610	1.53	**0.000**	0.85 (0.66-1.11)	0.237
CA199 (>37 U/ml)	1.95	**0.000**	1.16 (0.89-1.51)	0.275	2.14	**0.000**	1.30 (0.98-1.72)	0.065	1.92	**0.000**	1.33 (1.03-1.72)	**0.028**
NLR (≥2.36)	2.34	**0.000**	1.13 (0.68-1.87)	0.648	2.56	**0.000**	1.53 (1.11-2.11)	**0.010**	2.10	**0.000**	1.38 (1.04-1.82)	**0.025**
dNLR (≥1.85)	2.12	**0.000**	1.42 (1.08-1.87)	**0.012**	2.23	**0.000**	1.10 (0.66-1.85)	0.713	1.92	**0.000**	1.08 (0.69-1.69)	0.722
PLR (≥132)	1.51	**0.001**	1.03 (0.78-1.35)	0.858	1.56	**0.001**	0.96 (0.71-1.28)	0.763	1.42	**0.003**	0.94 (0.72-1.23)	0.674
LMR (≥4.95)	0.49	**0.000**	1.00 (0.73-1.35)	0.977	0.44	**0.000**	1.00 (0.71-1.40)	0.995	0.55	**0.000**	0.98 (0.73-1.32)	0.907

HR, hazard ratio; CI, confidence interval; NLR, neutrophil count to lymphocyte count; dNLR, neutrophil count to (white cell count – neutrophil count); PLR, platelet count to lymphocyte count; LMR, lymphocyte to monocyte ratio.

^a^Grade 1 and 2 stand for high or middle differentiated tumor, grade 3 and 4 stand for poorly differentiated tumor.

^b^Tumor stage according to seventh edition of American Joint of Committee on Cancer.

### Prognostic nomograms for CSS and DFS

**Figure 7:**
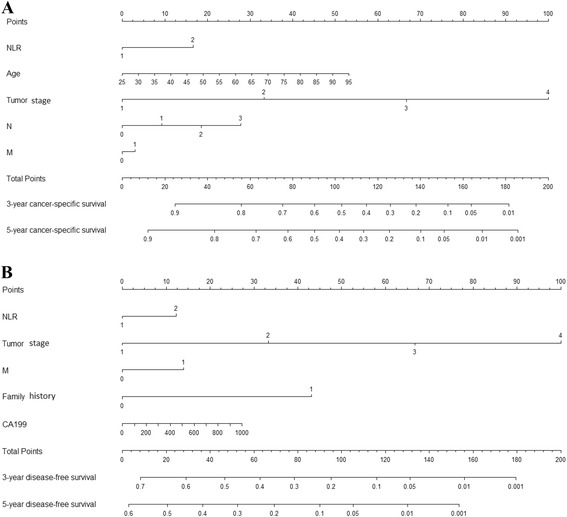
**Postoperative nomogram with NLR, dNLR and significant clinicopathologic characteristics predicted the probability of gastric cancer for cancer-specific survival (A) and disease-free survival (B).** To use the nomogram, an individual GC patient’s value is located on each variable axis, and a line is depicted upward to determine the number of points received for each variable value. Subsequently the sum of these numbers is located on Total Points axis, and a line is drawn downward to the survival axes to determine the likelihood of 3- or 5-year survival for CSS or DFS.

To predict the survival of GC patients after surgical resection, prognostic nomograms were depicted by Cox regression model analysis using all the significant independent indicators for CSS and DFS (Figure 7). These nomograms can predict the probability of recurrence or death for GC patients within 3 or 5 years after initial surgery, assuming the patient does not die of another cause first. The c-index for CSS and DFS prediction were 0.89 and 0.85, respectively.

## Discussion

The present study showed that pre-operation NLR, dNLR, PLR and LMR in the peripheral blood of GC patients were significantly associated with tumor progression and poor prognosis after surgical resection. Despite substantial progress in the understanding of the association between NLR, dNLR, PLR, LMR and the prognosis for cancers [11,21,27-29], previous studies only focused on the influence of one or two biomarkers on prognosis. This study was the first attempt to evaluate the prognosis of patients with GC based on these four biomarkers and to establish a predictive nomogram to improve the predictive accuracy.

The relationship between inflammation and cancer was first reported by Rudolf Virchow in 1863, who suggested that “lymphoreticular infiltrate” reflected the origin of cancer at the sites of chronic inflammation [6]. Over the past decades, Virchow’s hypothesis was verified by emerging evidence showing the influence of inflammatory microenvironment on cancer. More understanding of the links between inflammation and cancer contributes to the prevention and treatment of tumor. Consequently, several biomarkers have been reported to reflect the link between inflammation and cancer, such as interferon-gamma/interleukin-4 ratio [28] and inflammation-based prognostic score (Glasgow prognostic score) based on CRP and albumin levels [30]. Previous studies have shown that the systemic inflammatory biomarkers (NLR, dNLR, PLR, and LMR) can be also considered as potential prognostic factors for different types of carcinoma [24,31-33]. This study also indicated that OS, CSS, and DFS were obviously reduced in the high NLR, dNLR and PLR groups, but were decreased in the low LMR group. In multivariate analysis, dNLR showed a significant association with OS, and NLR was closely associated with CSS and DFS. The above results were supported by several mechanisms of inflammatory reaction to tumor.

Firstly, elevated neutrophils can secrete large amounts of reactive oxygen species (ROS), which induce cell DNA damage and genetic instability, causing both carcinogenesis and promotion in tumor microenvironment [34]. Elevated circulating neutrophils have been particularly reported to contain and secrete pro-angiogenic factors, including vascular endothelial growth factor (VEGF). Circulating VEGF contributes to tumor angiogenesis and the progression of neoplasm [35,36]. VEGF was observed to be significantly overexpressed in gastric cancer tissue compared with normal tissue [37], and it has been verified that the overexpression of VEGF can induce cell proliferation and promoted cell cycle of GC cells by increasing the activation of VEGF receptor 2 in vitro [38]. On the other hand, neutrophilia was triggered by cancer-related inflammatory factors, including tumor necrosis factor-alpha, interleukin-6, granulocyte colony stimulating factor, and myeloid growth factors [39-42].

Secondly, the decreased lymphocyte count and function play important roles in inflammatory reaction to tumor. It has been reported that neutrophil could inhibit T cell activation through the production of nitric oxide, arginase, and ROS [43], leading to depletion of lymphocyte-mediated immune response. This can explain why increased NLR may act as an independently prognostic factor for CSS due to elevated neutrophil and relative lymphocytopenia [44]. Previous study has proven that the density of tumor-associated macrophages in various cancers is correlated with increased angiogenesis, tumor invasion, and poor prognosis [45]. Tumor-associated macrophages derived from circulating monocytes are selectively released to tumor microenvironment by locally surged chemokines [4]. Thus, the circulating level of monocytes shows a surrogate for the formation or presence of tumor-associated macrophages.

In the present study, the optimal cut-off levels of NLR, dNLR, PLR and LMR calculated by ROC curves based on CSS were quite similar to those on the basis of OS and DFS, indicating that CSS could also be applied to calculate the optimal cut-off levels of NLR, dNLR, PLR and LMR. Indeed, some recent studies have employed CSS as an end-point to achieve the optimal cut-off point [24-26]. In the present study, it was observed that the optimal cut-off value of 2.36 for NLR was a superior prognostic level based on HR, which had the highest AUC and the best sensitivity and specificity. However, the optimal cut-off level for NLR in this study was inconsistent with the results of previous studies [11,20,21], which may be due to the differences in assays measuring neutrophil and lymphocyte, different population and different survival end-point for NLR [13,46].

Some nomograms have been developed in various cancers [47-49], and nomograms have shown to be more accurate than the conventional staging systems for predicting prognosis in cancers [50,51]. The present study attempts to establish a predictive nomogram to predict the probability of postoperative patients who will relapse and die of GC within 3-year and 5-year based on NLR, dNLR and clinicopathological factors. The nomograms performed well in predicting CSS and DFS, and their predictions were supported by c-index (0.89 and 0.85, respectively). These results supported that the nomograms could better predict prognosis in patients with GC post operation.

Some limitations of this study should be acknowledged. Firstly, the study was a retrospective design, with a small population size of 389 patients, which lead to no association between histological types and clinical prognosis. Secondly, the peripheral blood findings were not compared to the findings of peritumoral inflammation in the primary tumor tissue. Nevertheless, the peripheral blood results provide a novel horizon to understand the roles of NLR in carcinogenesis. Finally, there was some heterogeneity in the treatment used for patients after surgical resection, which led to different clinical prognosis. Hence, further studies are needed to illuminate the relationship between inflammatory biomarkers and prognosis in patients with GC.

## Conclusions

Preoperative NLR, dNLR, PLR and LMR are significantly associated with poor prognosis. Moreover, dNLR and NLR are independent prognostic indicators for OS and DFS, respectively, and NLR is a superior independent prognostic factor for CSS in surgical patients with GC. Therefore, integrate NLR, dNLR and the predictive nomogram could be used to evaluate prognosis and offer optimal therapeutic strategy.
